# Assessing genetic architecture and signatures of selection of dual purpose Gir cattle populations using genomic information

**DOI:** 10.1371/journal.pone.0200694

**Published:** 2018-08-02

**Authors:** Amanda Marchi Maiorano, Daniela Lino Lourenco, Shogo Tsuruta, Alejandra Maria Toro Ospina, Nedenia Bonvino Stafuzza, Yutaka Masuda, Anibal Eugenio Vercesi Filho, Joslaine Noely dos Santos Goncalves Cyrillo, Rogério Abdallah Curi, Josineudson Augusto II de Vasconcelos Silva

**Affiliations:** 1 Faculdade de Ciências Agrárias e Veterinárias, Universidade Estadual Paulista “Júlio de Mesquita Filho”, Jaboticabal, Sao Paulo, Brazil; 2 Animal and Dairy Science, Animal Breeding and Genetics, University of Georgia, Athens, Georgia, United States of America; 3 Instituto de Zootecnia, Nova Odessa, Sao Paulo, Brazil; 4 Instituto de Zootecnia, Sertãozinho, Sao Paulo, Brazil; 5 Faculdade de Medicina Veterinária e Zootecnia, Universidade Estadual Paulista “Júlio de Mesquita Filho”, Botucatu, Sao Paulo, Brazil; Kunming Institute of Zoology, Chinese Academy of Sciences, CHINA

## Abstract

Gir is one of the main cattle breeds raised in tropical South American countries. Strong artificial selection through its domestication resulted in increased genetic differentiation among the countries in recent years. Over the years, genomic studies in Gir have become more common. However, studies of population structure and signatures of selection in divergent Gir populations are scarce and need more attention to better understand genetic differentiation, gene flow, and genetic distance. Genotypes of 173 animals selected for growth traits and 273 animals selected for milk production were used in this study. Clear genetic differentiation between beef and dairy populations was observed. Different criteria led to genetic divergence and genetic differences in allele frequencies between the two populations. Gene segregation in each population was forced by artificial selection, promoting isolation, and increasing genetic variation between them. Results showed evidence of selective forces in different regions of the genome. A total of 282 genes were detected under selection in the test population based on the fixation index (Fst), integrated haplotype score (iHS), and cross-population extend haplotype homozygosity (XP-EHH) approaches. The QTL mapping identified 35 genes associated with reproduction, milk composition, growth, meat and carcass, health, or body conformation traits. The investigation of genes and pathways showed that quantitative traits associated to fertility, milk production, beef quality, and growth were involved in the process of differentiation of these populations. These results would support further investigations of population structure and differentiation in the Gir breed.

## Introduction

Gir is one of the main *Bos indicus* cattle breeds native from India that was first introduced in Brazil in late 1800s. Although Gir is an important breed for milk production in tropical South American countries, small populations are still found in North America and Australia. Few animals (<700) were imported in Brazil between 1870 and 1962. In 1938, the Brazilian Association of Zebu Cattle Breeders (ABCZ) started the registration of Gir cattle [1] and contributed to the dissemination of this breed in Brazil. In the 1960s, a number of Brazilian breeders started selecting for dual-purpose (milk and meat), and others selected only for milk production. Since 1993, the majority of the Gir breeders have been selecting mainly for milk yield [1].

Because of the importance for livestock and conservational systems, along with the reduced cost of whole-genome genotyping, genomic studies in Gir have become more common in the latest years. Most of the recent studies in Gir cattle are related to genetic structure, genetic diversity and inbreeding levels based on pedigree data in dairy herds [1], whole genome sequencing [2,3] for studying regions under selection for environmental adaptation [4], and genetic differentiation compared with other breeds [5]. Another study in a Gir population identified low levels of taurine introgression during the formation of this breed [6]. These studies were relevant for understanding the Gir history and exploiting the genetic background of the population. In 2013, three selective sweeps were identified and overlapped with the *ST6GALNAC5* gene in Gir [7]. Recently, Peripolli et al. (2018) [8] reported 14 runs of homozygosity (ROH) islands in a Brazilian Gir dairy population, including the animals used by Utsunomiya et al. (2013) [7]. ROH islands are regions of the genome with high levels of homozygosity and have been used to assess regions under selective pressure.

Strong artificial selection through domestication resulted in increased diversity among recent cattle populations. Diversity includes variation in morphology, physiology, production, and fertility [9]. Assessing patterns of genetic variation is of particular interest for studying domestication, breed formation, population structure, and consequences of selection [10]. Several measures of genetic differentiation have been proposed, and one of them is the fixation index (Fst; Wright (1951) [11]). Several studies have used Fst as a tool for identifying patterns of genetic variation at a locus among populations relative to that within populations. Thus, Fst has also been used as a test for identifying signatures of selection in populations [11, 12] by using high-throughput SNP information. In fact, Fst is one of the most commonly used metrics for detecting signatures of selection in animals.

Besides Fst, haplotype-based methods have also been used in signatures of selection studies. The integrated haplotype score (iHS) proposed by Voight et al. (2006) [13] and the cross-population extend haplotype homozygosity (XP-EHH) test proposed by Sabeti et al. (2007) [14] can help to spot evidence of selection with high detection power. Recombination has the power to break down linkage disequilibrium around a mutation, decreasing the length of haplotypes on which a mutation is located [15]. Sabeti et al. (2002) [16] introduced the extended haplotype homozygosity (EHH) to exploit the decay of haplotype homozygosity as a function of genetic distance from a focal SNP. Later, iHS was proposed as a complementary approach to the EHH method and is based on the standardized log ratio of the integrals of the observed decay of EHH computed for the ancestral and the derived alleles at the focal SNP [13]. The iHS test has been currently applied for detecting selection signatures in humans [15], plants [17], and domestic animals [4, 18, 19]. The XP-EHH is also based on the EHH method and is used to detect selection footprints between populations by comparing their EHH profiles.

The Fst and the haplotype-based approaches are somehow different. The time scale over which selection has been occurred has a major impact on the ability of each method to detect evidence of selection. The Fst method is best suited for detection in events occurring in the more distant past [20] whereas the iHS test is best suited for detection in recent positive selection [15]. The XP-EHH is useful for detection of entirely or approximately fixed loci [14]. As the Fst, iHS, and XP-EHH approaches are complementary, incorporating such measures in the study of genetic differentiation and selection would strongly contribute to understanding gene flow and genetic makeup of dual-purpose (meat and milk production) Gir cattle populations.

So far, a limited number of studies have been conducted to identify population structure and signatures of selection in Gir. O’Brien et al. (2015) [6] sampled bulls that were widely used for artificial insemination in dairy breeding systems, reflecting only the top animals in the breed. A study based on the iHS test did not detect significant numbers of signatures of selection in the breed probably because very few individuals were used (N = 53). Only the *ST6GALNAC5* gene was found harboring evidence of selection in Gir [7]. A sample of animals selected for different purposes from production herds across the country would be useful to understand genetic differentiation and selection footprints in the Brazilian Gir populations.

In this study, we present a comprehensive analysis of population structure and signatures of selection in two populations of Gir cattle selected either for beef or milk production, using high-throughput genomic information. Three methods (Fst, iHS, and XP-EHH) were implemented to scan the whole genome of those two populations. Afterwards, we performed a functional study of the genes identified within the regions harboring signatures of selection in each population to explain the biological importance of selection footprints. The information from our research can be useful for future GWAS studies, conservation, and genetic improvement of the Gir breed.

## Material and methods

### Data resource

The experiment was conducted in accordance with animal welfare guidelines according to State Law No. 11.977 of the São Paulo state, Brazil. All animal procedures were approved by the Ethics and Animal Handling Committee of the Instituto de Zootecnia, Nova Odessa, SP, Brazil.

Genotyped animals from two distinct populations were used in this study. A set of 273 female Gir were obtained from the Brazilian Program of Dairy Gir Genetic Improvement (PNMGL), which is a breeding program. These animals belonged to five farms located in Minas Gerais and Sao Paulo states. More details of the history and genetic background of this population can be found in Santana Jr et al. (2014) [1]. A set of 173 males and females were obtained from the Animal Science Institute (IZ; Sertaozinho, SP), which started a research program for growth traits in indicine breeds in 1976, including Gir.

### Differentiation between dairy and beef populations

Among all the Gir breeding or research programs in Brazil, two are of special interest: a) PNMGL aims improving milk production traits; b) IZ, which is considered unique, aims improving beef production traits in a closed herd scheme. The PNMGL was created in 1985 to conduct progeny testing programs [1], where the main objective was to identify genetically superior bulls for milk production, based on progeny performance. Traits such as milk production, milk composition (fat and protein), somatic cell counts, handling (e.g., ease of milking and temperament), and body conformation (e.g., ligament last udder, rear udder height, rear udder width, and length and diameter of the teats) are included in the breeding program. The mean 305-day milk yield of the current dairy Gir population is 3000 ± 1500 kg [1]. Because of the well-designed breeding program, semen of genetically proven bulls from PNMGL are commercialized worldwide. Recently, genomic information started being used in genetic evaluation of the Brazilian Gir cattle.

The IZ started the first breeding season in 1976 with different lines that were introduced in the Gir herd to increase genetic variability and avoid problems with inbreeding. Since the first breeding season, only animals from this herd were used for mating, making it a closed herd. The choice for founders was based on their yearling body weight (YBW) at 550 days. The first genetic evaluation was performed in 1981 [21] and the selection criterion for sires was the final body weight at 378 days of age, after 168-day feedlot performance tests. Females remained on pasture, and the selection criterion for females was their YBW at 550 days of age. For this herd, the generation interval was 5.65 years, and genetic gains were 2.88 kg/year for the weight of sires after the weight gain test and 2.80 kg/year for female considering the adjusted weight at 550 days [22]. Records of milk production were not available in this herd, animals were selected for growth traits.

### SNP array data and quality control

Animals used in this study were genotyped with the GGP Bovine LDv4 (Illumina, San Diego, CA) with approximately 33K SNP. Only SNPs located in the 29 autosomes remained for the analysis. Quality control was performed to ensure high quality of genomic data. Therefore, SNPs were removed when they were monomorphic, had a call rate lower than 0.90, and had a minor allele frequency (MAF) lower than 0.01, and when the difference between expected (Hardy-Weinberg equilibrium) and observed allele frequencies at a given locus was greater than 0.15. Samples with a call rate lower than 0.90 were also removed. After the quality control, a total of 442 animals and 23,275 SNPs were retained for further analyses. Filtering the SNPs based on MAF may affect the probability of identifying alleles related to selection [23]. For this reason, a low threshold for MAF (<0.01) was imposed as a criterion for the SNP quality control.

Imputation of missing SNPs and phasing were performed via Beagle 3.3.2 [24]. The phased data were annotated with ancestral reference alleles, and then the haplotype file and the physical map were used for the iHS analysis.

### Genetic structure of the population

To access the Gir population structure and to understand the relationship within and between populations in a genomic level, Principal Component Analysis (PCA) and Discriminant Analysis of Principal Components (DAPC) were performed using the adegenet package for the R software [25]. The PCA approach allows classifying individuals based on the reduced number of important orthogonal principal components (PC) [26]. Each PC relates its eigenvalue that describes the amount of total inertia explained on the component. When used in the context of genomic analysis of population structure, the eigenvalues indicate part of the total genetic variability represented by the associated PC. The first PC related to a high amount of inertia reproduces the structuring of the genetic data [27]. To perform PCA with different subsets of SNP markers, the default options for the *glPCA* function were used, allowing compensating for differences in variance among allele frequencies [27].

The DAPC was applied to select the optimal number of clusters (K), among all output clusters from PCA. The choice of K was made on the basis of the lowest associated Bayesian Information Criterion (BIC; Jombart et al. (2010) [25]). When the optimal number of clusters is ambiguous, K increases as long as it resulted in a noticeable improvement in BIC. DAPC uses K-means clustering of PC in order to infer the actual number of populations [25]. The DAPC was run using 100 PC and 10 discriminant functions.

### Divergent selection between populations

To verify the genetic divergence between two populations, genetic statistics such as observed heterozygosity (Ho), genetic diversity within population (Hs), total genetic diversity (Ht), coefficient of inbreeding (Fis), and fixation index (Fst) were computed using the HierFstat R package [28]. The fixation index (Fst), as defined in Nei (1987) [29], was used as a measure of genetic differentiation between populations being derived from the equation:

$$Fst\;=\;\frac{Ht-Hs}{Ht}$$

Fst quantifies differences in allele frequencies between populations, and theoretically its value ranges from zero to one, meaning that there is no differentiation or complete differentiation in which subpopulations are fixed for different alleles, respectively. Fst values are effective for identifying selection signatures between different groups, i.e., loci in which alleles are fixed differently in different groups [30] and allow determining how the divergent selection can affect the genomic pattern of these groups [31].

As a way to compare Fst values for each SNP, measures of centrality and dispersion such as mean and standard deviation were considered. The negative Fst values observed for 2,794 SNPs were set to zero, since negative values have no biological interpretation [32]. Loci were plotted relative to their physical position within each autosome. The threshold to call an SNP outlier was defined as three standard deviations above the mean. This methodology allows identifying SNPs with Fst values that stand out from the others, and that could be related to genes affecting adaptive and/or economical important traits for beef or milk production, meaning evidence of selection signatures. Similar approaches have been used in other studies for identifying selection signatures [12, 31]. A control chart approach allows the partition of Fst variation into a component due to selection and also allows the discovery of significant SNPs based on control limits set at three standard deviations from the mean [12]. Kijas et al. (2012) [10] and Zhao et al. (2015) [31] used the top 0.1% Fst values to represent selection signature.

The approach adopted in the present study is a simplistic technique that allows to access the variation patterns on many loci and considers values above the cut-off as evidence of divergent selection. Assuming that the data follow a normal distribution, a Fst value above three standard deviations from the mean indicate that the locus has a value higher than 99.8% of total SNPs in the chromosome.

### Within population test

The iHS test was used to detect strong footprints of selection within the studied population. This test is based on the standardized log ratio of integrals of the observed decay of extended haplotype homozygosity (EHH), computed for both ancestral and derived alleles at the focal SNP.

Phased haplotypes produced by Beagle 3.3.2 and ancestral allele information were submitted to iHS-based tests. For each SNP, ancestral and derived alleles were determined according to the 50K SNP annotation file provided in Rocha et al. (2014) [33], which included information of putative ancestral alleles of bovine SNPs. The number of SNPs in common between the GGP LDv4 SNP chip and the 50K SNP was 26,058. The phased haplotypes were annotated with ancestral allele information (via R package rehh 2.0 [15]). For each allele in dairy and beef populations, information was defined as missing if the code was incompatible with ancestral and derived alleles. The iHS score was computed for each autosomal SNP using the R package rehh 2.0 [17]. Default options were generally used, except for the minimal threshold on the minor allele frequency that was set to 0.01 and the percentage of retained haplotypes that were changed to suit the Gir haplotype data. In addition, the standardization of iHS was performed with allele frequency bins of 0.01, which is controlled by the ihh2ihs function in the package [15].

According to Voight et al. (2006) [13], the iHS of a given focal SNP (iHS) was defined as

$$iHS\;=\;\frac{UniHS-\;\mu_{UniHS}^{p_{s}}}{\sigma_{UniHS}^{p_{s}}}$$

where UniHS = log(iHH_ancestral_/iHH_derived_) (where iHH is the integrated allele-specific extended haplotype homozygosity for core SNP alleles (ancestral and derived)), and $\mu_{UniHS}^{p_{s}}$ and $\sigma_{UniHS}^{p_{s}}$ are the average and the standard deviation of the UniHS, respectively, computed over all the SNPs with a derived allele frequency p_s_ similar to that of the core SNPs. The iHS was constructed to approximately follow a standard Gaussian distribution and to enable comparisons among SNPs regardless of their underlying allele frequencies. The iHS is transformed into piHS, as shown by [34]:

$$p_{i}HS\;=\;-{log}_{10}(1-2|\Phi (iHS)-0.5|)$$

where *Φ*(*x*) represents the Gaussian cumulative distribution function. Assuming that most of the iHS values follow the Gaussian distribution under neutrally, piHS may be interpreted as a two-sided *p*-value in a -log10 scale [15].

To control for false positives, the fndr.cutoff function available in the *fdrtool* R package [35] was used with its default options for calculating a *p*-value, which defines the cut-off point chosen according to the false non-discovery rate. After the false discovery rate (FDR) adjustment within population, the genome-wide significance level was equal to approximately 0.006 and 0.008 for dairy and beef cattle populations, respectively. This methodology has been used to identify regions displaying strong footprints of selection in humans [15], cattle [31, 36] and other animal populations [18].

### Cross-population test

To compare EHH profiles between the two populations, we used the XP-EHH statistics. The main idea of XP-EHH is to test if the genome site is homozygous in one population but polymorphic in another population through the comparison of EHH score of two populations on one core SNP [37]; ancestral information is not needed when performing this test. In our analysis, the genome of the beef population, as an observed population, was compared with the dairy population, as a reference population. The XP-EHH score was computed for each autosomal SNP using the same R package used to compute iHS, rehh 2.0 [15].

The XP-EHH of a given focal SNP (XP-EHH) was defined and standardized, according to Sabeti et al. (2007) [14] and Gautier et al. (2017) [15] as

$$XP-EHH\;=\;\frac{LRiES-{med}_{LRiES}}{\sigma_{LRiES}}$$

where LRiES = log(iEH_population1_/iEH_population2_); iEH is the integrated allele-specific extended haplotype homozygosity; *med*_LRiES_ and *σ*_LRiES_ are the median and the standard deviation of the LRiES, respectively. Populations 1 and 2 are considered as a reference population and an observed population, respectively. The XP-EHH is transformed into *p*_*XP*-*EHH*_ as shown by Gautier et al. (2011) [34]:

$$p_{XP-EHH}\;=\;-{log}_{10}(1-2|\Phi (XP-EHH)-0.5|)$$

where *Φ*(*x*) represents the Gaussian cumulative distribution function. As *p*_*XP*-*EHH*_ may be interpreted as a two-sided *p*-value in a -log10 scale, regions with a *p*-value lower than 0.01 (1%) were considered signatures of selection in the test populations. Negative XP-EHH scores suggest selection happened in the reference population, otherwise happened in the observed population.

### Candidate genes and functional analysis

A genomic region was considered as being under selection if it contained significant SNPs based on Fst or iHS values. Windows of 500 kb (250 kb upstream and 250 kb downstream of the significant SNP) were investigated to verify overlapping gene segments. Additionally, genes were compared with QTL regions previously identified and present in the Cattle QTL database (https://www.animalgenome.org/cgi-bin/QTLdb/BT/search). Gene annotation was performed using the UMD3.1 bovine genome assembly from the BioMart (www.ensembl.org/biomart) and NCBI (https://www.ncbi.nlm.nih.gov) databases. The orthologous genes from primates and sheep were used when the annotation information for bovine genes was not available.

A database for Annotation, Visualization, and Integrated Discovery (DAVID) v6.8 tool [38, 39] was used to identify significant (p < 0.05) Gene Ontology (GO) terms and KEGG (Kyoto Encyclopedia of Genes and Genomes) pathways using a list of genes with significant SNPs based on Fst and iHS values and the *Bos taurus* annotation file as a reference genome.

## Results and discussion

**Fig 1:**
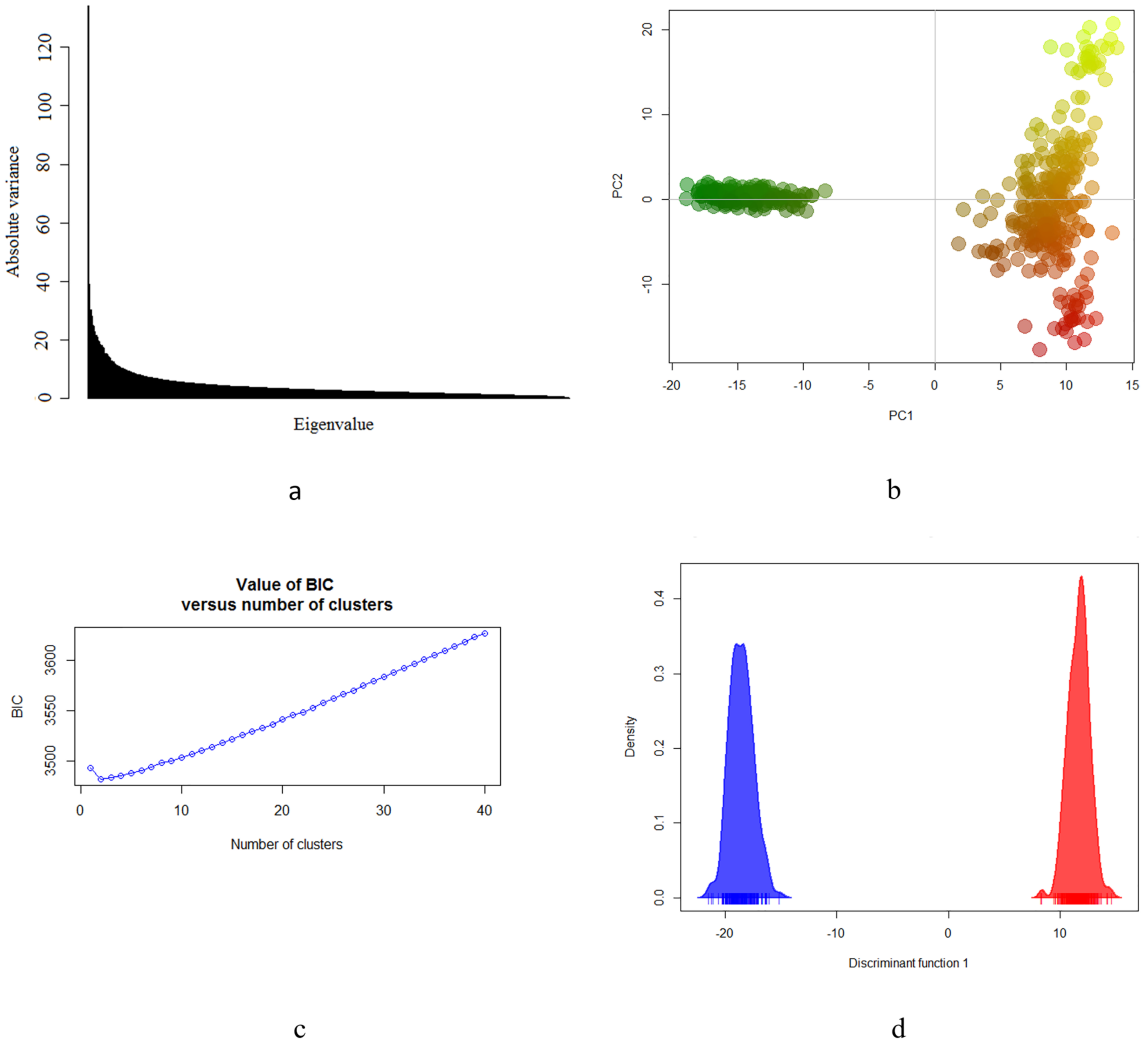
Genetic structure of the Brazilian Gir population. (a) Eigenvalues of principal component analysis (PCA). (b) PCA scatter-plots of the first two principal components (PC) showing clearly separation between beef population (green color) and dairy population (red-yellow colors). (c) Inference of the number of clusters in Gir cattle based on K-means algorithm. (d) Plots of the first two discriminant functions of discriminant analysis of PC algorithm.

Eigenvalues of the PCA are shown in Fig 1a. The genetic structure of the dataset, i.e. the variance of the data, was captured mainly by the first PC. The eigenvalue for the first PC was 134.11, and the eigenvalue for the second PC was 39.16 (Fig 1a). Although eigenvalues are absolute variances of the corresponding PC, it is common to express them as a percentage of the total variation in the data. In this case, percentages of variance explained by first and second PCs were 6.79 and 1.98, respectively. The percentage of the variance explained by PC was calculated as the eigenvalue times 100 divided by the sum of all the eigenvalues.

In Fig 1b, it is clear that both groups were completely separated according to the first principal component (PC1). The obtained results are clear and consistent with the history of both populations, which were under different breeding programs; therefore, were subjected to the intentional segregation of genes within each population, promoting the complete isolation and genetic variation between them. The dairy population presented a more scattered cluster based on the second component (PC2), indicating higher genetic distance and lower relatedness between individuals of the population, compared with the beef population cluster. Because animals from the dairy population were from commercial herds located in different regions of Brazil, it is possible that the PCA analysis clustered individuals of the dairy population according to their different demographic histories, providing a more scattered pattern. The proximity between animals from the beef population could be due to the fact that it was characterized as a closed herd in which only breeding animals of this herd were used in the mating. Mating of related animals favored the increase of inbreeding and reduced the genetic difference among individuals of this group.

Results of PCA were based on the clustering of individuals into geographically pre-defined groups. Discriminant Analysis of Principal Components based on the K-means algorithm, implemented in the R package adegenet [25] was further used to test the greatest number of clusters and to better evaluate the hypothesis on the two populations. An inspection of BIC values when the number of clusters (K) ranged from 1 to 100 showed that two clusters should be considered (Fig 1c); given this was the number with the lowest BIC. This test reflects the minimum number of clusters after which the BIC increases or decreases by a negligible amount [25]. The estimation of K by the DAPC has been markedly successful for the inference of the exact number of clusters in recent population genetic studies [40, 41]. The plots of the first discriminant function of DAPC with different colors for different groups provided a visual assessment of between-population genetic structure (Fig 1d). In the present study, DAPC can also be considered well suited to describe the genetic diversity of the genotyped animals in the Brazilian Gir population.

**Fig 2:**
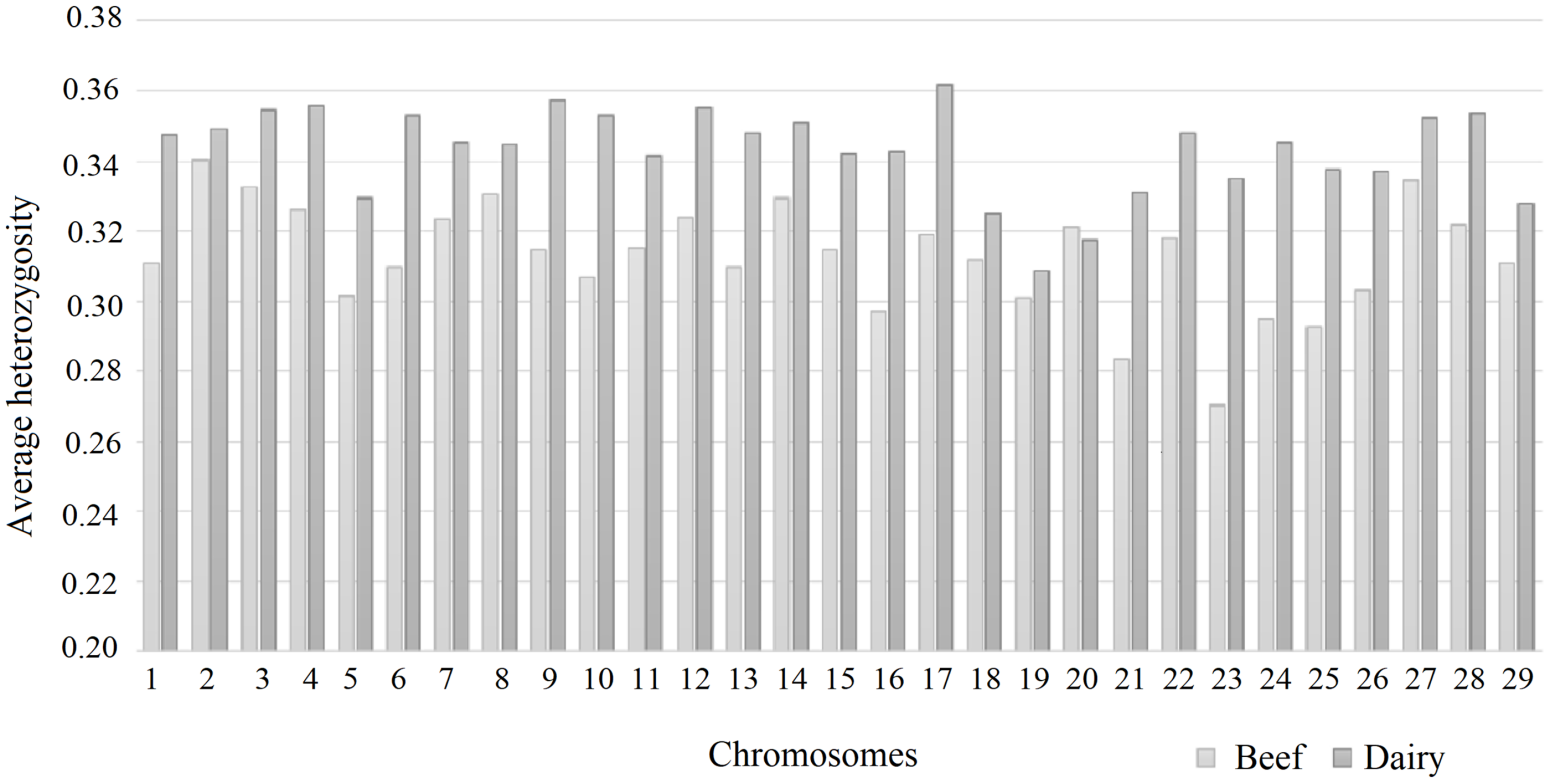
Average heterozygosity per chromosome in two populations. Light grey = beef cattle; dark grey = dairy cattle.

According to PCA and DAPC analyses, there was a clear genetic difference between beef and dairy populations, and this division reflects the variation existent between the populations. Because of that, the next step was to compute genetic statistics taking into account the stratification of the dataset into beef and dairy populations. Average heterozygosity by chromosome was different between the two populations (Fig 2). The greatest discrepancy between means was observed on chromosome 23 (0.06). The allele frequency-dependent diversity estimate, such as observed heterozygosity (H_o_), is a measure of genetic variation and can be very useful in comparison between populations [10]. The dairy population displayed higher heterozygosity levels compared with the beef population, which most likely reflects relative levels of genetic diversity. The highest heterozygosity level was observed on chromosome 17 (0.38), and the exception was for chromosome 20, where the beef population displayed the higher mean. Fis was slightly negative for dairy (-0.008) and beef (-0.0322) populations. Negative values of Fis could indicate an excess of heterozygosity beyond that expected under Hardy-Weinberg equilibrium within those populations. Negative values of Fis in Gir and other cattle breeds have been reported in the literature [6, 42].

**Fig 3:**
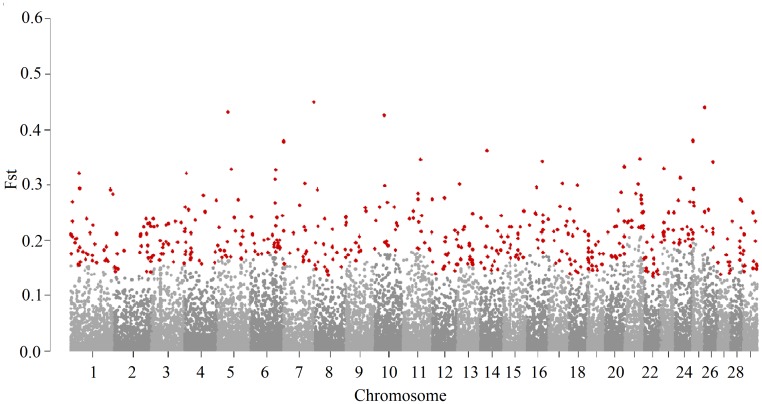
Genomic distribution of Fst values. SNPs were plotted relative to their physical positions within each autosome. The cutoff to call SNP outliers was defined as three standard deviations above the mean for each autosome. Red dots are SNPs with Fst beyond the cutoff value.

Genetic differentiation varied throughout the genome (Fig 3). The average value for Fst was 0.033. Although the two populations in the present study were selected for different purposes, some levels of proximity between them were expected and were indicated by the low Fst values. In addition, the average Fst value was close to that observed in different cattle breeds [9, 30].

The level of genetic differentiation between populations is expected to be low in neutral regions of the genome or in regions of balanced selection, and divergent in regions subject to directional selection. When a Fst value is zero, there is no genetic differentiation between the populations under comparison. In the present study, low Fst values were possibly identified because the two populations belonged to the same breed and were originated from the same recent ancestral population; therefore, many alleles were expected to be commonly fixed in both populations. Gir is originally a dual-purpose breed. During the 1960s, part of the Brazilian breeders were artificially selecting animals for meat and milk simultaneously [1], which could explain the expected levels of proximity and low Fst values for the two populations. These populations only started being selected for different purposes in the last decades. Under artificial selection, allele frequencies can shift over time in the direction of the desired phenotypes producing signatures of selection.

**Fig 4:**
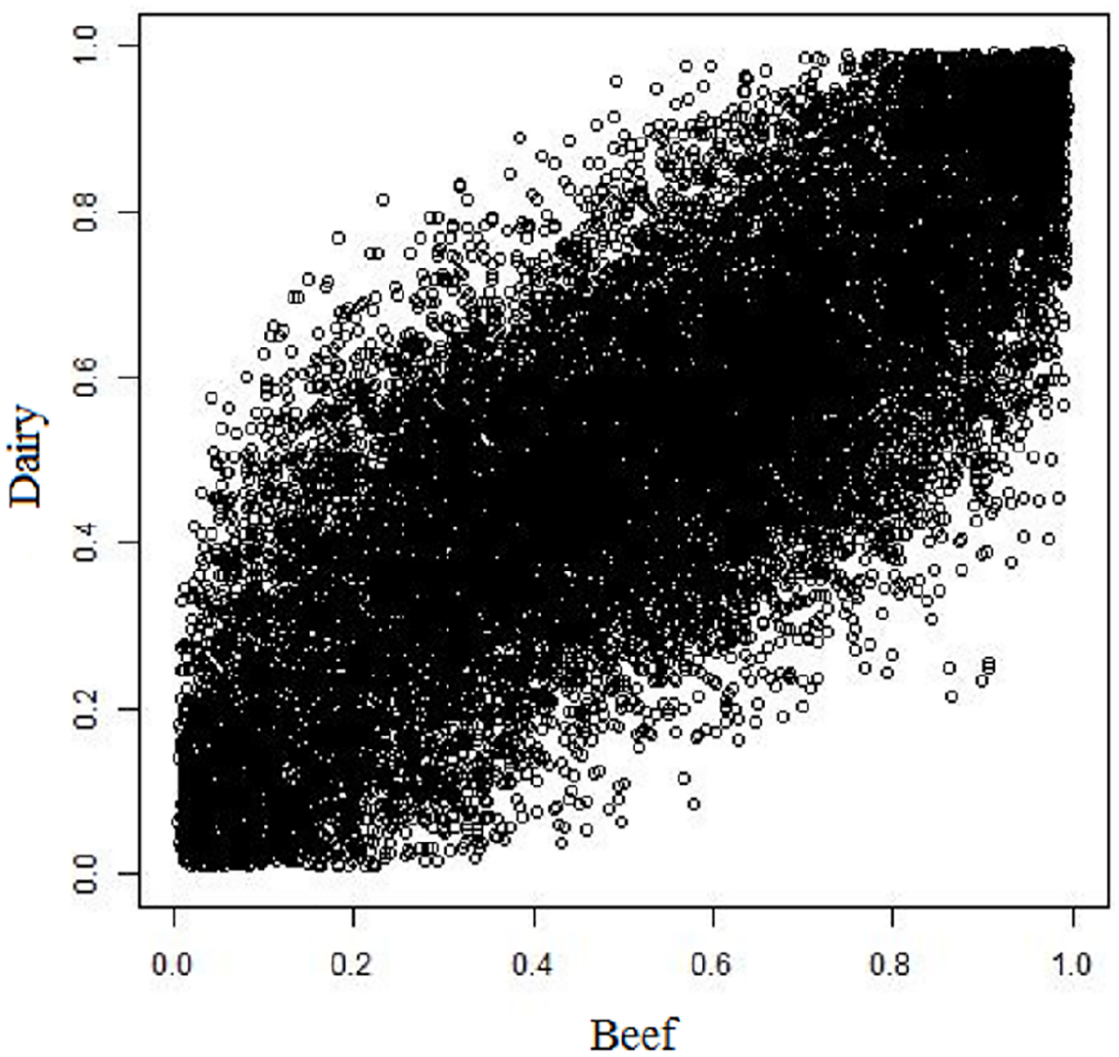
Scatter plots for population-specific allele frequency (dairy x beef). The data were displayed as a collection of points; each point represents an SNP having the allele frequency for the dairy population determining the position on the vertical axis and the allele frequency for the beef population determining the position on the horizontal axis. It reveals a positive linear relationship between allele frequencies of two populations.

The Fst approach enables the detection of selection signatures based on differences in allele frequencies across populations. This method revealed 488 SNPs as relevant loci for divergent selection among the total number of loci evaluated. Although being from the same breed, some changes in allele frequencies were observed between the two populations (Fig 4). This change in allele frequencies and the genetic diversity observed were possibly caused by recent selection for different criteria and other non-reported genetic events that occurred in the past. These findings are supported by the population structure results. The higher allele frequencies of these SNPs are representative of differences in selection, neutrality or other processes used in breeding programs [40].

The SNPs identified as outliers based on the Fst method can be strong evidence of signatures of selection. Four SNPs had Fst values higher than 0.4; they were located on chromosomes 5, 7, 10, and 26. In total, 19 SNPs had Fst values ranging from 0.3 to 0.4. The greatest number of outliers was obtained on chromosome 1 (n = 33, with Fst values ranging from 0.15 to 0.32) and the lowest was obtained on chromosome 25 (n = 7, with Fst values ranging from 0.20 to 0.38) (S1 Fig).

Considering the threshold of three standard deviations to report an outlier Fst, the SNPs elected as outliers had Fst values higher than 99.8% of the total SNPs in the chromosome, which identified a variation pattern within the chromosome. Cadzow et al. (2014) [20] acknowledged that distinct methods use different patterns of genetic variation to identify evidence of selection. In addition, they highlighted the importance of utilizing multiple methodologies for investigating selection among populations. Thus, the iHS test was used as a complementary approach to identify regions that exhibit evidence of selection. This is a linkage disequilibrium-based method that provides increased power for assessment of signatures of selection within the population using high-throughput molecular information (e.g., SNP arrays).

**Fig 5:**
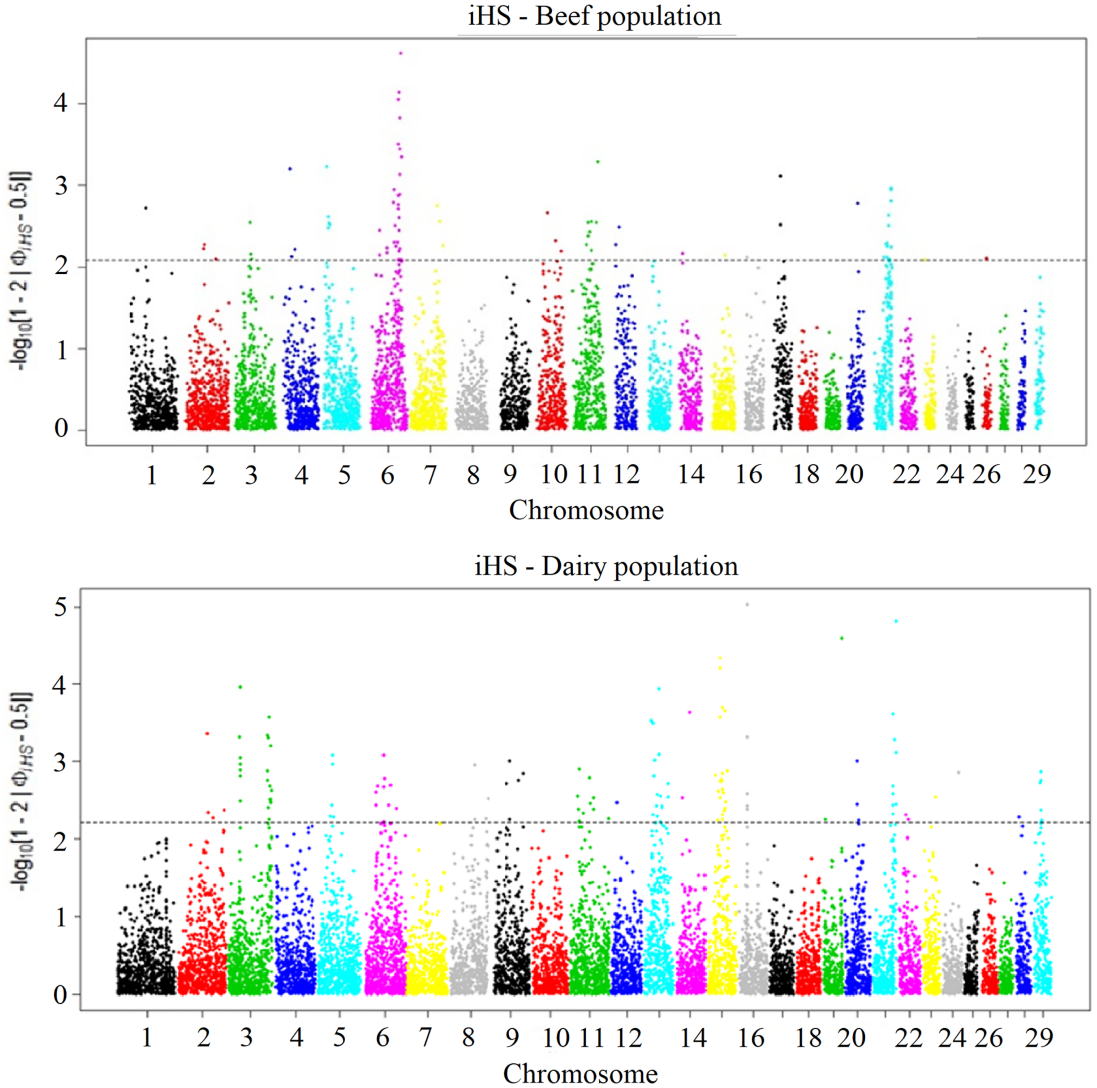
Genome-wide distribution of |iHS| values for Gir cattle. Upper = beef population; bottom = dairy population.

After adjustment for FDR within the population, SNPs displaying piHS values greater than or equal to 2.10 (which approximately corresponded to p-value <0.008) were considered significant for the beef population. For the dairy population, the cutoff value was 2.20 (which approximately corresponded to p-value <0.006). The "fdrtool" package allows the computation of local FDR values from p-values while taking into account an empirical null model [35]. The application of the FDR adjustment within each population avoids the detection of false positive selection signatures [31]. The chromosome-wide scans of iHS for beef and dairy populations are shown in Fig 5. The plots show clear evidence of selective forces in different regions of the genome. A total of 82 and 129 SNPs harbored signatures of selection in beef and dairy populations, respectively. The most significant SNP mapped to chromosome 6 (95,171,308 bp) for the beef population (iHS = -4.22) and to chromosome 16 (25,933,132 bp) for the dairy population (iHS = -4.43).

To further identify regions displaying strong signatures of selection, 500 kb windows containing the significant SNPs were investigated. A total of 23 and 43 candidate genes overlapped with significant windows for beef and dairy populations, respectively. The description of the top ten most significant iHS genomic regions per population is shown in Table 1. The *SLC24A4* (solute carrier family 24 member 4) gene located on chromosome 21 was common in the two populations, which can be clearly confirmed by the iHS plots (Fig 5). It has been shown that the *SLC24A4* gene belongs to a group of the potassium-dependent sodium/calcium exchanger proteins which has been associated with hair, skin and eye pigmentation in humans [43, 44]. Nayeri et al. (2016) [45] conducted a genome-wide association study (GWAS) and identified the *SLC24A4* gene in a significant region associated with success in fertility traits such as calving interval and days open in female Canadian dairy Holstein cattle.

**Table 1:** Top ten significant iHS genomic regions harboring signatures of selection in beef and dairy Gir cattle.

Population	Position^a^	Peak position	iHS^b^	piHS^c^	Candidate gene (position)
**Beef**	Chr4:35784894–35785394	35785144	-3.42	3.20	*SEMA3D* (35563538–35793762)
	Chr5:4589482–14589982	14589732	3.44	3.23	*SLC6A15* (14572186–14629483)
	Chr6:88541997–88542497	88542247	-3.92	4.05	*n*.*a*
	Chr6:88936051–88936551	88936301	-3.61	3.51	n.a.
	Chr6:90771212–90771712	90771462	3.97	4.14	*LOC101906736*^d^ (90743814–90771489)
	Chr6:93023933–93024433	93024183	3.57	3.45	*CCDC158* (92975081–93058373)
	Chr6:93906353–93906853	93906603	3.79	3.82	n.a.
	Chr6:95171058–95171558	95171308	-4.22	4.62	n.a.
	Chr6:98735539–98736039	98735789	-3.51	3.34	n.a.
	Chr11:75228982–75229482	75229232	-3.47	3.29	*ATAD2B* (75141063–75263986)
**Dairy**	Chr3:33004500–33005000	33004750	3.86	3.95	n.a.
	Chr13:41168962–41169462	41169212	-3.85	3.93	n.a.
	Chr14:41835997–41836497	41836247	3.68	3.63	n.a.
	Chr15:37760348–37760848	37760598	4.00	4.20	*LOC104974236*^d^ (37754671–37769288)
	Chr15:37871231–37871731	37871481	4.08	4.34	n.a.
	Chr15:44711168–44711668	44711418	3.72	3.70	*STK33* (44548054–44742390)
	Chr15:52440542–52441042	52440792	-3.69	3.65	n.a.
	Chr16:25932882–25933382	25933132	-4.43	5.03	*DUSP10* (25895748–25936856)
	Chr19:56552331–56552831	56552581	-4.21	4.59	*RECQL* (556549762–56580017)
	Chr21:66999575–67000075	66999825	4.32	4.81	*WDR25* (66917744–67066001)

n.a., not available.

^a^ All positions are given in base pairs (bp) according to the Bovine UMD3.1 assembly.

^b^ iHS is a value of integrated haplotype score.

^c^ piHS is a value of transformed integrated haplotype score.

^d^ non-coding RNA.

The description of the top ten most significant XP-EHH genomic regions per population is shown in Table 2. The most significant SNP was mapped to chromosome 22 (58,886,462 bp) for the beef population (XP-EHH = 2.44) and to chromosome 21 (57,721,222 bp) for the dairy population (XP-EHH = -7.02). We detected 28 SNPs as candidates of selection footprints in the beef population when the dairy population was used as a reference in the XP-EHH test, but only 10 out of 28 SNPs were mapped in gene regions. For the beef population, seven genes (*DNAH7*, *FBLN2*, *HECW2*, *SLC8A2*, *TRRAP*, *UGT1A1*, *and WNT7A*) were identified based on the XP-EHH statistics. Two SNPs were located in the region of the *UGT1A1* gene, and two SNPs were located in the region of the *WNT7A* gene. The *UGT1A1* gene was located on chromosome 3 and encodes a critical enzyme that transforms small lipophilic molecules, such as steroids, hormones, and drugs, into excretable metabolites maintaining homeostasis [46]. The *WNT7A* gene regulates several cells and developmental pathways that affect the development of female reproductive tract and maintain uterine function in adults [47]. The *WNT7A* gene enhances muscle regeneration, increases the satellite stem cells and stimulates myogenic stem cell motility [48, 49]. Xue et al. (2013) [50] identified polymorphisms in the WNT7A gene associated with growth traits in Chinese Qinchuan cattle.

**Table 2:** Top ten significant XP-EHH genomic regions harboring signatures of selection in beef and dairy Gir cattle.

Population	Position^a^	Peak position	XP-EHH^b^	pXP-EHH^c^	Candidate gene (position)
**Beef**	Chr5:62492901–62493401	62493151	2.20	1.56	n.a.
	Chr15:80280407–80280907	80280657	2.24	1.60	n.a.
	Chr18: 54862897–54863397	54863147	2.33	1.71	*SLC8A2* (54853503–54881796)
	Chr18: 54955789–54956289	54956039	2.19	1.55	n.a.
	Chr19:11998622–11999122	11998872	2.22	1.58	n.a.
	Chr19:12009008–12009508	12009258	2.29	1.66	n.a.
	Chr22:58836201–58836701	58836451	2.28	1.65	*WNT7A* (58809372–58873170)
	Chr22:58886212–58886712	58886462	2.44	1.83	n.a.
	Chr22:58896176–58896676	58896426	2.22	1.58	n.a.
	Chr25:38067748–38068248	38067998	2.17	1.53	n.a.
**Dairy**	Chr21:57531747–57532247	57531997	-6.66	10.56	n.a.
	Chr21:57532846–57533346	57533096	-6.72	10.75	*SLC24A4* (57596461–57783306)
	Chr21:57619270–57619770	57619520	-6.64	10.51	*SLC24A4* (57596461–57783306)
	Chr21:57619838–57620338	57620088	-6.67	10.61	*SLC24A4* (57596461–57783306)
	Chr21:57720972–57721472	57721222	-7.02	11.65	*SLC24A4* (57596461–57783306)
	Chr21:57723114–57723614	57723364	-6.90	11.29	*SLC24A4* (57596461–57783306)
	Chr21:57723895–57724395	57724145	-6.84	11.11	*SLC24A4* (57596461–57783306)
	Chr21:57726974–57727474	57727224	-6.79	10.96	*SLC24A4* (57596461–57783306)
	Chr21:57729925–57730425	57730175	-6.81	11.02	*SLC24A4* (57596461–57783306)
	Chr21:57730971–57731471	57731221	-6.65	10.54	*SLC24A4* (57596461–57783306)

n.a., not available.

^a^ All positions are given in base pairs (bp) according to the Bovine UMD3.1 assembly.

^b^ XP-EHH is a value of cross-population extend haplotype homozygosity score.

^c^ pXP-EHH is a value of transformed cross-population extend haplotype homozygosity score.

For the dairy population, 655 negative values presented p-values higher than 0.01, but only 197 out of 655 outliers were mapped to gene regions. When searching for genes, only 94 were identified for the dairy population. The *SLC24A4*, *DUSP10*, *CTNNA2*, *ADAMTS3*, and *TTC12* genes were located in regions with the highest number of SNPs identified per window, being 29, 12, 9, 7, and 7 SNP per region, respectively. The *SLC24A4* were also detected with iHS for the beef and dairy population, and the *DUSP10* gene was also detected with iHS approach. The *CTNNA2* gene was detected with the Fst, iHS and XP-EHH approaches.

The *DUSP10* gene located on chromosome 16 negatively regulates the activation of mitogen-activated protein (MAP) kinases and has a principal function in both innate and adaptive immune responses as an inhibitor of inflammation [51]. This gene has been described playing an important role in regulating the balance of energy thought the control of brown adipocyte differentiation [52]. Huang et al. (2017) [53] comparatively analyzed the transcriptome of subcutaneous adipose tissue between Wagyu and Holstein breeds with difference in fat deposition and identified the *DUSP10* gene up-regulated in Wagyu, which could be a key gene associated with fat metabolism and adipogenesis. The *CTNNA2* (catenin alpha 2) gene codifies a protein that plays an important role in the catabolism of collagen, cell adhesion and myogenesis [54]. This gene was identified in a QTL region associated with Lipomatous Myopathy in Piedmontese beef cattle, a disease characterized by degeneration and infiltration of the muscular tissue characterized by replacement of myofibers with adipose tissue [55].

The *ADAMTS3* gene was mapped on chromosome 6 and encodes a member of the procollagen aminopropeptidase subfamily of proteins that play a role in the processing of type II fibrillar collagen in articular cartilage which is crucial for embryonic development and regulates placental angiogenesis [56]. Mészáros et al. (2014) [57] performed a genome-wide association study in Fleckvieh bulls and identified the *ADAMTS3* gene associated with longevity.

In total, 157 out of 488 significant Fst values were located in 151 regions. Three SNPs with significant Fst values overlapped with the *FCHSD2* (FCH and double SH3 domains 2) gene, which is located on chromosome 15 and regulates cell growth, migration and adhesion [58]. Two Fst peaks were within the genomic regions of *NSG1* (neuronal vesicle trafficking associated 1), *RALGPS1* (Ral GEF with PH domain and SH3 binding motif 1), and *HS3ST5* (heparan sulfate-glucosamine 3-sulfotransferase 5) genes, which are located on chromosomes 6, 11 and 9, respectively.

The *NSG1* gene encodes a small transmembrane protein highly expressed specifically in neurons which plays a critical role in the trafficking and polarization of several proteins [59–61]. Lee et al. (2016) [62] reported the *NSG1* gene as a region containing highly selective SNPs, i.e. SNPs that have higher probability of being selected in the next generation for milk production traits (milk yield and fat and protein contents) in Holsteins.

The *HS3ST5* gene encodes a member of the heparan sulfate 3-O-sulfotransferases protein family that catalyzes the biosynthesis of heparan sulfate, which regulates blood coagulation [63]. This gene has been associated with reproductive seasonality in sheep (total days of anoestrus and progesterone cycling months) [64].

Three genes (*CTNNA2*, *SLC24A4*, and T*MEM117*) were detected based on more than one approach. In the dairy population, three genes (*CNTN3*, *STK33*, and *TMEM117*) were detected with Fst and iHS, four genes (*DUSP10*, *NCAM1*, *SLC24A4*, and *TMEM117*) were detected with iHS and XP-EHH, and thirteen genes (*AFP*, *ANKRD17*, *CSNK1G1*, *C4orf22*, *CTNNA2*, *GALNT14*, *MAP1A*, *TBC1D22B*, *TMEM117*, *TMPRSS11E*, *UNC79*, *VPS13C*, and *ZFAND3*) were detected with Fst and XP-EHH methods. Eight genes (*ADAMTS3*, *BCL11A*, *CAPN13*, *CTNNA2*, *EPHA5*, *GC*, *SLC24A4*, and *SLC25A21*) were detected based on the iHS for the beef population and XP-EHH for the dairy population.

The *CNTN3* (contactin 3) gene located on chromosome 22 has been described as a candidate gene for tenderness in Nelore beef cattle [65]. The *STK33* (serine/threonine kinase 33) gene located on chromosome 15 encodes a member of the calcium/calmodulin-dependent kinase family, which exhibits a non-ubiquitous and a low level of expression in most tissues [66]. The *TMEM117* (transmembrane protein 11) located on chromosome 5 codifies an uncharacterized multi-pass transmembrane protein. Veerkamp et al. (2012) [67] performed a GWAS for feed utilization complex in Holstein–Friesian dairy cows and identified an SNP in *TMEM117* gene with a large effect in body condition scores. In another study also with GWAS, Zhu et al. (2017) [68] identified this gene associated with saturated fatty acids composition in Simmental cattle. The *NCAM1* was mapped on chromosome 15 and plays important functional roles in cell migration and plasticity changes in the developing and adult nervous system [69], insulin signaling and adipocyte differentiation in mouse [70].

A total of 282 genes were detected under selection in the dairy and beef Gir populations based on Fst, iHS, and XP-EHH approaches (see S1 File for details). Chen et al. (2016) [9] did not find common candidate regions in Chinese Holstein and Simmental cattle populations by using Fst and XP-EHH methods. The authors attributed it to different features of these methods and recommended the integration of various methods to increase the detection sensitivity of signatures of selection. As Fst, iHS, and XP-EHH tests assume different methodologies, more confidence is provided when a common genomic region is obtained by various methods. Fst, iHS, and XP-EHH must be used as complementary approaches in the detection of signatures of selection.

We compared the gene list from each test with previous regions of selection signatures in Gir. Three significant SNPs harboring signatures of selection based on the iHS were identified for Gir dairy cattle by Utsunomiya et al. (2013) [7]; only one of the SNPs identified by the authors was located in the intergenic region of the *ST6GALNAC5* gene.

The *ST6GALNAC5* gene was not identified in our analysis. However, six genes identified in our study (*ARHGEF7*, *DNAH7*, *EPB41*, *RELL1*, *TRAPPC9* and *ZFHX4*) were located within the ROH islands reported by Peripolli et al. (2018) [8] in Brazilian Gir dairy cattle. The ROH islands are genomic regions with high homozygosity around a selected locus, i.e., with reduced genetic diversity. These islands of the genome might harbor targets of positive selection.

Next, we compared our gene list with QTL regions previously reported in cattle and available for online search. In total, 35 genes with signatures of selection overlapped with QTL regions. Mapping *LCORL* and *NCAPG* genes to the cattle QTL database showed that both genes were associated with reproduction, growth, and meat and carcass traits. They are all located on chromosome 6. A number of QTL terms have been reported for cattle in the region of *NCAPG* and *LCORL* genes [71, 72]. The *LCORL* and *NCAPG* were identified based on iHS test in the dairy population.

Other genes with selection signatures that had overlapped with QTL terms were found being associated with production (*KCNIP4*, *ANXA4*, *FTO*, *EGFR*, and *PAK1*), reproduction (*VAV3*, *KCNIP4*, *FRAS1*, *C4orf22*, *TRAPPC9*, and *APBB1*), milk composition (*ROBO1*, *NRCAM*, *KCNIP4*, *DAAM1*, *TRAPPC9*, *NF2*, and *FTO*), meat and carcass (*FAM184B*, *NCAM1*, *NF2*, *CTSD*, *TOX*, and *VSTM2L*), health (*TMTC2*, *IL1RN*, *BFSP1*, *TOX*, *CNTN3*, and *TRAPPC9*), and body conformation (*GC*, *ANXA4*, and *NF2*). More details can be found in the supplementary S1 File. The above findings suggest that these genes might play an important role for traits that have been selected in both dairy and beef populations.

The dataset used for DAVID v.6.8 comprised 142, 23, 43, and 94 genes identified based on Fst, iHS beef, iHS dairy, and XP-EHH dairy tests, respectively. Regarding to the XP-EHH beef test analysis, only seven genes (*DNAH7*, *FBLN2*, *HECW2*, *SLC8A2*, *TRRAP*, *UGT1A1*, *and WNT7A*) were identified in those genomic regions, which is not suitable for a functional analysis.

The significant (P<0.05) GO terms (biological process, cellular component, and molecular function) and KEGG pathways identified with Fst, iHS dairy, and XP-EHH dairy tests are described in Table 3. No significant terms were detected to the iHS beef cattle analysis, probably due to the small number of genes on this dataset. Results from the functional enrichment analysis with the Fst test revealed a total of six KEGG pathways, four GO cellular components, six GO biological processes, and one GO molecular function (Table 3).

**Table 3:** Gene Ontology terms and KEGG pathways enrichment analysis.

Test	Term	P	Genes
**Fst**	***KEGG Pathway***		
	bta04530:Tight junction	0.0150	*MAGI3*, *EPB41*, *ACTN1*, *AMOTL1*, *TJP2*
	bta04810:Regulation of actin cytoskeleton	0.0160	*EGFR*, *ARHGEF4*, *VAV3*, *ARHGEF7*, *DIAPH3*, *ACTN1*
	bta04970:Salivary secretion	0.0200	*KCNMA1*, *PLCB4*, *ADCY9*, *PRKG2*
	bta04540:Gap junction	0.0240	*EGFR*, *PLCB4*, *ADCY9*, *PRKG2*
	bta00240:Pyrimidine metabolism	0.0332	*NME5*, *PRIM2*, *POLR3A*, *POLR2D*
	bta00230:Purine metabolism	0.0340	*NME5*, *ADCY9*, *PRIM2*, *POLR3A*, *POLR2D*
	***Gene Ontology Cellular Component***		
	0045211~postsynaptic membrane	0.0072	*KCNMA1*, *GRIK2*, *CLSTN1*, *NSG1*, *HTR3B*
	0030027~lamellipodium	0.0103	*CORO1C*, *NF2*, *ARHGEF7*, *ACTN1*, *AMOTL1*
	0005925~focal adhesion	0.0105	*CORO1C*, *EGFR*, *SYNE2*, *ARHGEF7*, *LPP*, *GIT2*, *ACTN1*, *SYNPO2*
	0016328~lateral plasma membrane	0.0287	*CORO1C*, *NSG1*, *ACTN1*
	***Gene Ontology Biological Process***		
	0019228~neuronal action potential	0.0151	*KCNMA1*, *GRIK2*, *SCN5A*
	0035023~regulation of Rho protein signal transduction	0.0224	*ARHGEF4*, *VAV3*, *ARHGEF7*, *ARHGEF10L*
	0030032~lamellipodium assembly	0.0240	*ARHGEF4*, *VAV3*, *ARHGEF7*
	0045184~establishment of protein localization	0.0240	*CORO1C*, *SMYD3*, *MCC*
	0030036~actin cytoskeleton organization	0.0306	*CORO1C*, *NF2*, *DIAPH3*, *DAAM1*
	0042384~cilium assembly	0.0486	*CEP295*, *NME5*, *TTBK2*, *AHI1*
	***Gene Ontology Molecular Function***		
	0050518~2-C-methyl-D-erythritol 4-phosphate cytidylyltransferase activity	0.0147	*ISPD*
**iHS dairy**	***Gene Ontology Cellular Component***		
	0097431~mitotic spindle pole	0.0174	*NUMA1*, *EML1*
	***Gene Ontology Biological Process***		
	0007420~brain development	0.0201	*EML1*, *RAB18*, *SLC6A17*
	***Gene Ontology Molecular Function***		
	0043140~ATP-dependent 3'-5' DNA helicase activity	0.0114	*RECQL5*, *ASCC3*
	0016887~ATPase activity	0.0166	*ABCG5*, *MACF1*, *CLPB*
	0005524~ATP binding	0.0247	*STK33*, *ABCG5*, *RECQL5*, *ASCC3*, *CLPB*, *PAK1*, *CPS1*
**XP-EHH dairy**	***KEGG Pathway***		
	***Gene Ontology Biological Process***		
	GO:0030326~embryonic limb morphogenesis	0.0107	*FRAS1*, *PBX1*, *ALX1*
	GO:0032964~collagen biosynthetic process	0.0250	*TRAM2*, *ADAMTS3*
	GO:0009952~anterior/posterior pattern specification	0.04771	*GRSF1*, *PBX1*, *ALX1*
	***Gene Ontology Molecular Function***		
	GO:0005096~GTPase activator activity	0.103	*RASAL2*, *ELMOD3*, *RALGAPA1*, *TBC1D22B*, *ARHGAP25*

The regulation of the actin cytoskeleton (bta04810) and tight junction (bta04530) are pathways from the cellular process class, related to cellular community and cell motility, respectively. The actin cytoskeleton is a dynamic skeletal cell structure that contains actin and associated proteins, which has been associated with important functions in several biological processes including early development of oocyte organization and maturation [73], tenderness of *Longissimus dorsi* muscle from Qinchuan cattle [74], *Longissimus dorsi* intramuscular adipose tissue in Hanwoo cattle [75], response to diet [76] and calf birth weight in Holstein cattle [77], and response to several pathogens such as *Trypanosoma congolense* [78], *Salmonella enterica* [79], and *Mycobacterium bovis* [80].

Tight junctions are essential for establishing a selectively permeable barrier to diffusion through the paracellular space between cells, which are composed of transmembrane proteins that are involved in junction assembly, barrier regulation, cell polarity, gene transcription, and among other pathways. Tight junctions are known to be related to milk mammary gland development and milk secretion, controlling the transport of lactose and K+ to the extracellular fluid, whereas Cl− and Na+ are transported into milk [81, 82]. Since decreasing in the tight junction permeability results in increasing milk secretion, the tight junction pathway (bta04530) has been reported involved in milk production and quality traits in dairy cattle [83–85].

The functional enrichment analysis with the XP-EHH dairy test results revealed one GO molecular function and three GO biological processes (Table 3). The GTPase activator activity (GO:0005096) is a molecular function related to GTPase-activating proteins (GAP), a family of regulatory proteins that can bind to activate G-proteins and stimulate its intrinsic GTPase activity. Regulation of G-proteins is important because these proteins are involved in important cellular processes and physiological functions [86]. Regarding to GO biological processes identified by the XP-EHH dairy test, all of them were involved in the embryonic development and specification of patterns of cell differentiation. The embryonic limb morphogenesis (GO:0030326) is a biological process that occur in the embryo by which the anatomical structures of the limp are generated and organized; the anterior/posterior pattern specification (GO:0009952) is defined as a regionalization process in which specific areas of cell differentiation are determined along the anterior-posterior axis (line that runs from the head to the tail of an organism), while the collagen biosynthetic process (GO:0032964) is related to chemical reactions and pathways resulting in the formation of collagen that is a group of proteins that form the main component of connective tissue in animals. Considering the intrinsic features of each test, genes identified by the iHS approach are potentially involved in recent selection pressures. Conversely, genes identified by the Fst method are likely related with events occurring further in the past. However, there is no guarantee that captured patterns of genotypic variation are a result of selection alone. It can be a result of other unrelated ancestral events [20] or even it may represent false positive results. To control the false positive rate, we applied the FDR adjustment within each population as recommended in the literature [31]. For the Fst, we used a technique similar to a control chart, but in a simplistic way that was based on measures of central tendency and dispersion.

Even though we used a tool available in the fdrtool R package [35] to control for false positives in the iHS test, it is difficult to completely rule out potential false positives. Teshima et al. (2006) [87] advised that error rates can be further decreased by combining several statistics. Even when a stringent cutoff of 1% for significance is defined, a fraction of loci identified as targets of selection may, in fact, be false discoveries [87]. In this way, it is possible that the target of selection that stood out in the Fst, iHS, and XP-EHH methods may contain a fraction of false discoveries. Genetic historical events different from artificial selection such as bottleneck, genetic drift, and migration could shape the population genomic variation [88, 89]. In addition to the effects of positive selection, genetic drift might be the other cause of genetic sweep altering the genetic structure of the two cattle populations. Further investigations need to be done to distinguish demographic history from selection in the two populations used in this study. Methods to compare models of positive selection relative to nonequilibrium models have been proposed by Jensen et al., 2007 [88] to accurately define the demographic events in a population.

The number of animals used in the present study is not a major concern in the structure and signatures of selection analysis. Several studies have focused on reduced number of animals per population, for example, <150 animals [18, 90] and <550 animals [7, 36]. This issue can be solved by the choice of appropriate methods among the large diversity of existing methods in the literature. As we have seen, the approaches used in the present study are largely recommended and powerful for comparison and studying genotypic variation in different populations.

The use of non-related animals in population structure studies is difficult in livestock species, although the choice for non-related animals allows for high genetic differentiation. For a small livestock population, the choice for animals to be genotyped are usually made by their importance in the herd. It is possible that the genotyped animals are related because breeding programs use tools as non-random mating systems and reproductive technologies, which can increase the inbreeding level in the population. In the case of Gir cattle, the level of inbreeding is high and the effective population size is small for the dairy [1] and the beef populations. We consider that each breeding program was well represented by the chosen animals. Further improvements for the present study can be probably achieved by using a greater variety of SNP in the genotyping panel.

We found evidence of signatures of selection for two Gir cattle populations artificially selected for different purposes (e.g., meat or milk production). The detection of selection signatures used here can act as complementary information to current gene mapping approaches (GWAS). By comparing candidate gene regions found through the identification of selection signatures and GWAS, it is possible to test the contribution of genes under selection to phenotypes, which can subsequently be used in genomic selection [9]. The findings of the present study provided preliminary details about the recent adaptation of Brazilian Gir cattle. The loci that we identified as selection signatures provided information about genes and pathways in which the two Gir populations have adapted to the selective pressures. Strong selection on each population led to specialization of these populations. As we have seen in the investigation of genes and pathways, traces associated with fertility, milk production, beef quality, and growth were involved in this process. Furthermore, these selective signals indicated the presence of genetic variants that must affect complex phenotypes of particular interest for conservation and genetic improvement of the Brazilian Gir cattle. As a whole, the results found here give basic support for further investigations in the Gir breed.

## Conclusion

High-throughput genomic information such as SNP markers can be successfully used to study the population structure and to identify genomic regions undergone divergent selection in Gir cattle. The difference in breeding history was able to imprint a degree of genomic differentiation for both beef and dairy populations. The patterns of genotypic variation in Gir cattle were consistent with the presence of selective pressures at some point in the history of the beef and dairy populations. These findings can provide complementary information on genomic regions of interest for functional genomic studies, genome-wide associations, and the implementation of breeding schemes aiming genetic improvement and conservation of livestock populations.

## Supporting information

S1 Fig Distribution of Fst values for chromosomes 1 and 25. (TIF)

S1 File Intergenic regions harboring signatures of selection in the Brazilian Gir cattle. (XLSX)
