# Supplementary figures and images for: Assessing genetic architecture and signatures of selection of dual purpose Gir cattle populations using genomic information
Source: PLoS One. 2018 Aug 2;13(8):e0200694. doi: 10.1371/journal.pone.0200694 (PMC6071998; doi:10.1371/journal.pone.0200694)

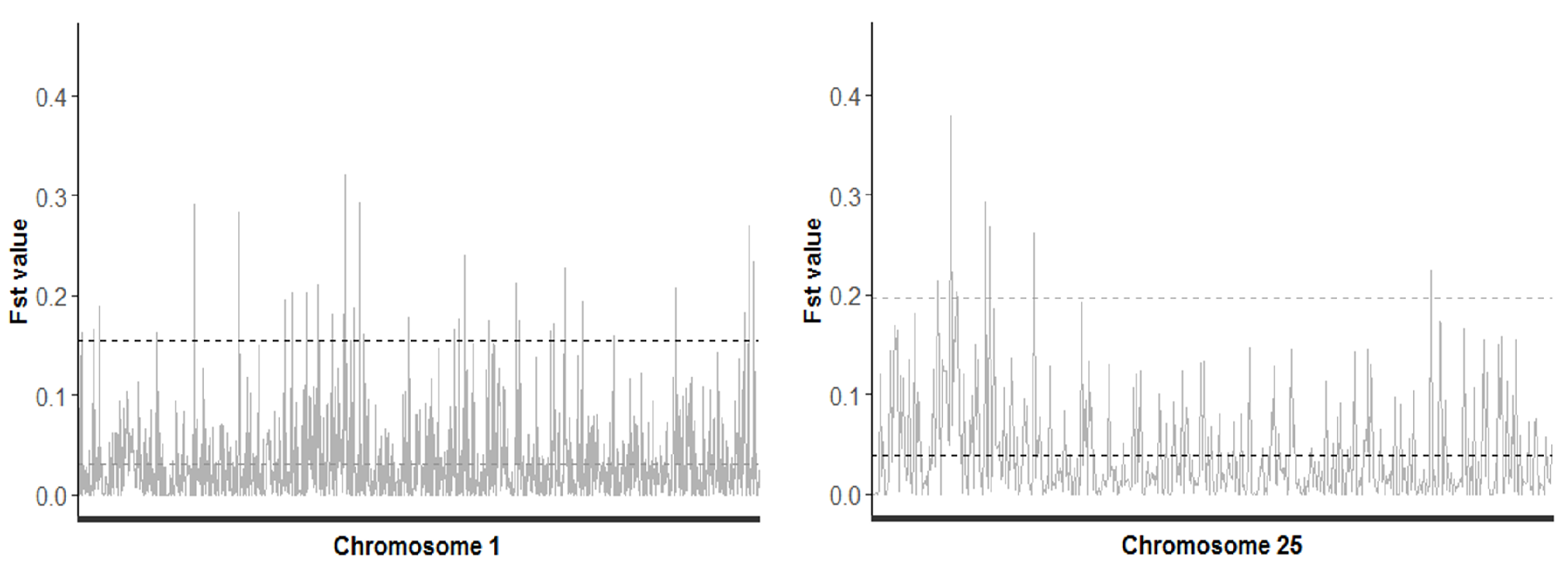

Supplement: S1 Fig — (TIF) [file pone.0200694.s001.tif]
